# Sublattice‐Resolved Magnetoelastic Coupling and Opposing Pressure Responses in the Frustrated Magnetocaloric GdMn_2_O_5_

**DOI:** 10.1002/advs.77490

**Published:** 2026-08-31

**Authors:** Romualdo S. Silva, João E. Rodrigues, Javier Gainza, Henrik L. Andersen, Federico Serrano‐Sánchez, Neven Biskup, Olivier Mathon, Norbert M. Nemes, José L. Martínez, José A. Alonso

**Affiliations:** ^1^ Instituto De Ciencia de Materiales de Madrid (ICMM), CSIC Madrid Spain; ^2^ European Synchrotron Radiation Facility (ESRF) Grenoble France; ^3^ Departamento De Física de Materiales Universidad Complutense de Madrid Madrid Spain; ^4^ Instituto Pluridisciplinar Universidad Complutense de Madrid Madrid Spain

**Keywords:** charge ordering, cryogenic refrigeration, frustrated magnetism, GdMn_2_O_5_, magnetocaloric effect, multiferroics, pressure tuning

## Abstract

Frustrated multiferroic manganites exhibit strong lattice‐charge‐magnetic coupling, enabling complex phase transitions and tunable magnetocaloric responses for cryogenic cooling. Here, we systematically investigate GdMn_2_O_5_ to elucidate its structure, magnetic ordering, magnetocaloric properties, and pressure response. Structural studies reveal an orthorhombic *Pbam* framework with no global phase transition from 4–300 K, but instead show anisotropic thermal expansion, including negative expansion along the *b*‐axis, driven by magnetoelastic coupling. Microscopy and X‐ray absorption spectroscopy confirm Mn^3+^/Mn^4+^ charge ordering between octahedral and pyramidal sites, with an effective coordination number of 5.5. The compliant Mn–Gd interface acts as the primary transducer of magnetoelasticity, exhibiting anomalous spin‐phonon coupling. Magnetic measurements reveal successive transitions: *T_N_* ≈ 39 K (Mn^3+^/Mn^4+^ antiferromagnetic ordering), *T_SR_* ≈ 34 K (spin‐reorientation), and *T_Gd_* ≈ 3.5 K (Gd^3+^ ordering). Under 0–7 T, the material displays a conventional magnetocaloric effect with a maximum entropy change of 16.1 J/kg K at ∼8 K and a relative cooling power of 272 J/kg, surpassing prior reports. Hydrostatic pressure reveals opposing responses: *T_N_* decreases, consistent with weakened Mn–O–Mn exchange, while *T_Gd_* increases, suggesting enhanced Mn–Gd coupling under compression. These results highlight GdMn_2_O_5_ as a promising cryogenic magnetocaloric material and reveal a sublattice‐dependent pressure‐tuning mechanism relevant to frustrated multiferroics.

## Graphical abstract

In GdMn_2_O_5_, sublattice‐resolved EXAFS and diffraction analysis reveal pronounced spin‐phonon coupling and an unexpected opposing pressure response: the Mn‐driven Néel temperature slightly decreases while the Gd‐sublattice ordering temperature rises under pressures up to 0.86 GPa. Combined with a high magnetocaloric refrigerant capacity, these results highlight GdMn_2_O_5_ as a promising, pressure‐tunable platform for cryogenic magnetic refrigeration.

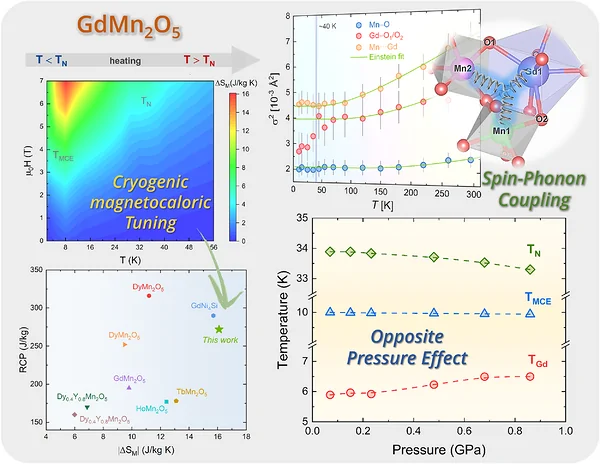

## 1 Introduction

Multiferroic materials, which simultaneously exhibit coupled ferroic orders such as ferroelectricity and antiferromagnetism, have attracted sustained interest for both fundamental physics and next‐generation spintronics [1, 2]. Among the most intensively studied families of multiferroics are the rare‐earth manganites of the general formula *R*Mn_2_O_5_ (*R* = rare earth, Bi, Y) [3, 4, 5]. These compounds adopt an orthorhombic crystal structure (space group *Pbam*, No. 55), characterized by a distinctive network of edge‐sharing Mn^4+^O_6_ octahedra and Mn^3+^O_5_ square pyramids. The spatial separation of Mn^3+^ and Mn^4+^ ions, coupled with frustrated magnetic interactions arising from the zig‐zag topology of the Mn sublattice, gives rise to complex magnetic phase diagrams that typically include multiple antiferromagnetic (AFM) transitions, spin‐reorientation phenomena, and magnetically induced ferroelectricity [6, 7].

The magnetocaloric effect (MCE) has been extensively studied in several benchmark material families, including pure rare‐earth elements (Gd, Dy, Ho), Heusler alloys (Ni‐Mn‐Ga), La(Fe_13‐_ *_x_* Si*_x_*)‐based alloys, layered double hydroxides based on transition metals (α‐Co(OH)_2_:Mo), MnAs‐based alloys, perovskite manganites (La_1‐_ *_x_* A*_x_* MnO_3_), double perovskites (R_2_NiMnO_6_), and spinel ferrites (Zn*_x_* Ni_1‐_ *_x_* Fe_2_O_4_) [8, 9]. In particular, the *R*Mn_2_O_5_ family displays an interesting MCE performance, with maximum magnetic entropy changes (−*ΔS_M_^max^*) typically ranging from 6 to 13 J/kg K [10, 11, 12, 13]. Among these, GdMn_2_O_5_ stands out as a promising yet underexplored candidate. Although previous studies have characterized its magnetic transitions and crystal structure, its magnetocaloric properties have received limited attention. The reported −Δ*S_M_^max^* value of approximately 9.8 J/kg K at 0–7 T [12] suggests that further enhancements are achievable through optimized synthesis routes and a deeper understanding of structure‐property correlations. Thus, unraveling the interplay between crystal structure and magnetic behavior is essential for the rational design of advanced functional multiferroic materials.

Magnetoelastic coupling plays a central role in connecting magnetic order to lattice distortions. In this context, we have recently demonstrated the critical role of structural‐magnetism features in optimizing MCE performance, achieving a −Δ*S_M_^max^* ≈ 21 J/kg K in Gd_2_CrSbO_7_ [14]. Furthermore, we extended these correlations to double perovskite oxides, establishing a framework for tuning magnetic properties through controlled structural modifications [15, 16, 17, 18]. Within this scenario, the gadolinium‐containing member GdMn_2_O_5_ occupies a particularly interesting position because the Gd^3+^ ion possesses an ^8^S_7/2_ ground state (*L* = 0, *J* = 7/2), which renders it essentially isotropic and insensitive to crystal electric field effects [19], an attribute that makes it an ideal probe for isolating the role of magnetoelastic coupling from single‐ion anisotropy. This electronic configuration distinguishes Gd^3+^ from other rare‐earth ions (e.g., Dy^3+^, Ho^3+^, Tb^3+^) that exhibit significant magnetic anisotropy due to unquenched orbital angular momentum. Consequently, in GdMn_2_O_5_, the rare‐earth sublattice contributes a purely spin magnetic moment, making it an ideal platform for investigating the interplay between Mn and Gd sublattices without the complicating influence of crystal‐field anisotropy [20]. Furthermore, the large magnetic moment of Gd^3+^ (*µ* ≈ 7.94 µ_B_) and its weak coupling to the crystal lattice suggest that Gd‐based oxides may exhibit substantial MCE, which is the physical principle underlying magnetic refrigeration [12, 21]. Moreover, under hydrostatic pressure, a new commensurate magnetic phase has been observed in several members of the family, which progressively replaces the ambient‐pressure incommensurate phases and is accompanied by a notable enhancement of Mn–Mn exchange striction [22]. This pressure‐induced phase stabilization results from the competition between the Mn–Mn super‐exchange interactions and rare‐earth magnetic anisotropy, offering a pathway to manipulate both magnetic ordering and MCE response in this multiferroic system [22, 23].

In this work, we comprehensively investigate the structural, electronic, magnetic, and magnetocaloric properties of polycrystalline GdMn_2_O_5_. Using synchrotron X‐ray diffraction with pair distribution function analysis (4–300 K) and atomic‐resolution STEM‐EELS, we reveal anisotropic thermal expansion, direct spectroscopic evidence of Mn^3+^/Mn^4+^ charge ordering, and the absence of a global structural phase transition. Magnetic measurements reveal two magnetic transitions (*T_N_* ≈ 39 K and *T_SR_* ≈ 34 K) followed by Gd^3+^ ordering at ∼3.5 K. To complement the long‐range structural characterization obtained by SXRD and provide site‐specific information on the local electronic structure of each sublattice, X‐ray absorption spectroscopy (XAS) measurements were performed at the Gd *L*_3_‐edge and Mn *K*‐edge: the Gd *L*_3_‐edge (2*p*_3/2_ → 5*d* transition) directly probes the valence state and coordination symmetry of the Gd^3^^+^ ion, while the Mn *K*‐edge (1*s* → 4*p* transition) yields quantitative evidence of Mn^3+^/Mn^4+^ charge ordering and local distortions of the [MnO_6_] octahedra and [MnO_5_] square pyramids. Our material exhibited a maximum magnetic entropy change of 16.1 J/kg K at *T_MCE_* ≈ 8 K under 0–7 T, with a relative cooling power of 272 J/kg, substantially exceeding previously reported values for GdMn_2_O_5_. Finally, quasi‐hydrostatic pressure (up to 0.86 GPa) reveals an opposing sublattice‐dependent response: *T_N_* decreases due to weakened Mn–O–Mn super‐exchange, while *T_Gd_* increases as Mn–Gd coupling is enhanced. These findings demonstrate GdMn_2_O_5_ as a promising cryogenic magnetocaloric material derived from relatively inexpensive and non‐toxic elements, while also providing chemical insight into sublattice‐selective pressure tuning in frustrated multiferroics.

## 2 Results

### 2.1 Crystalline Structure at Room Temperature

**FIGURE 1:**
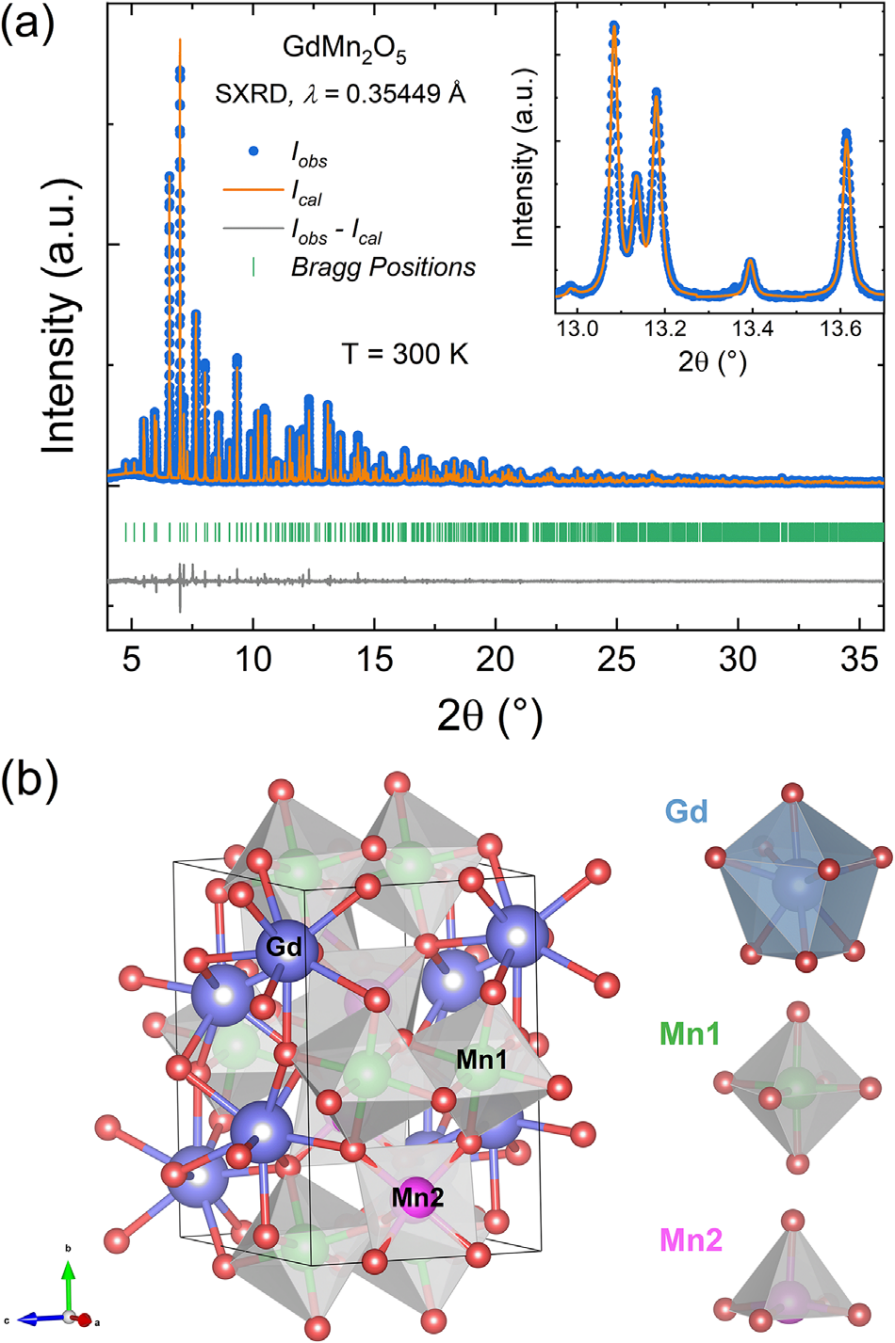
(a) Rietveld plot from SXRD data at room temperature for GdMn_2_O_5_. (b) 3D view of crystalline structure.

Figure 1a displays the experimentally obtained powder SXRD pattern at room temperature and corresponding Rietveld refinement profiles for the GdMn_2_O_5_ sample. All diffraction peaks can be well‐fitted by the orthorhombic structure in the *Pbam* space group (No. 55), yielding refined lattice parameters of *a* = 7.3582(1) Å, *b* = 8.5452(9) Å, and *c* = 5.6860(5) Å, in agreement with the literature [4, 12]. The resulting Rietveld refinement parameters are listed in Table S1. Figure 1b presents the schematic crystal structure of GdMn_2_O_5_. The unit cell consists of corner‐sharing (Mn_1_)^4+^‒O_6_ octahedra that form chains along the *c*‐axis. These chains are cross‐linked by (Mn_2_)^3+^‒O_5_ pyramids, which lie in the plane normal to the *c*‐axis. The O3 and O4 atoms connect the octahedra and pyramids along the *a*‐ and *b*‐axes, respectively, while O1 and O2 link pyramid pairs and octahedra along the *c*‐axis. The nearest <Gd‒O>, <Mn_1_‒O>, and <Mn_2_‒O> distances were found to be 2.397(8), 1.904(7), and 1.946(4) Å, whereas the <Mn_1_‒O‒Mn_1_>, <Mn_2_‒O‒Mn_2_>, and <Mn_1_‒O‒Mn_2_> bond‐angles were 95.14(1)°, 96.81(1)°, and 127.94(5)°, respectively.

### 2.2 Scanning Transmission Electron Microscopy Study

**FIGURE 2:**
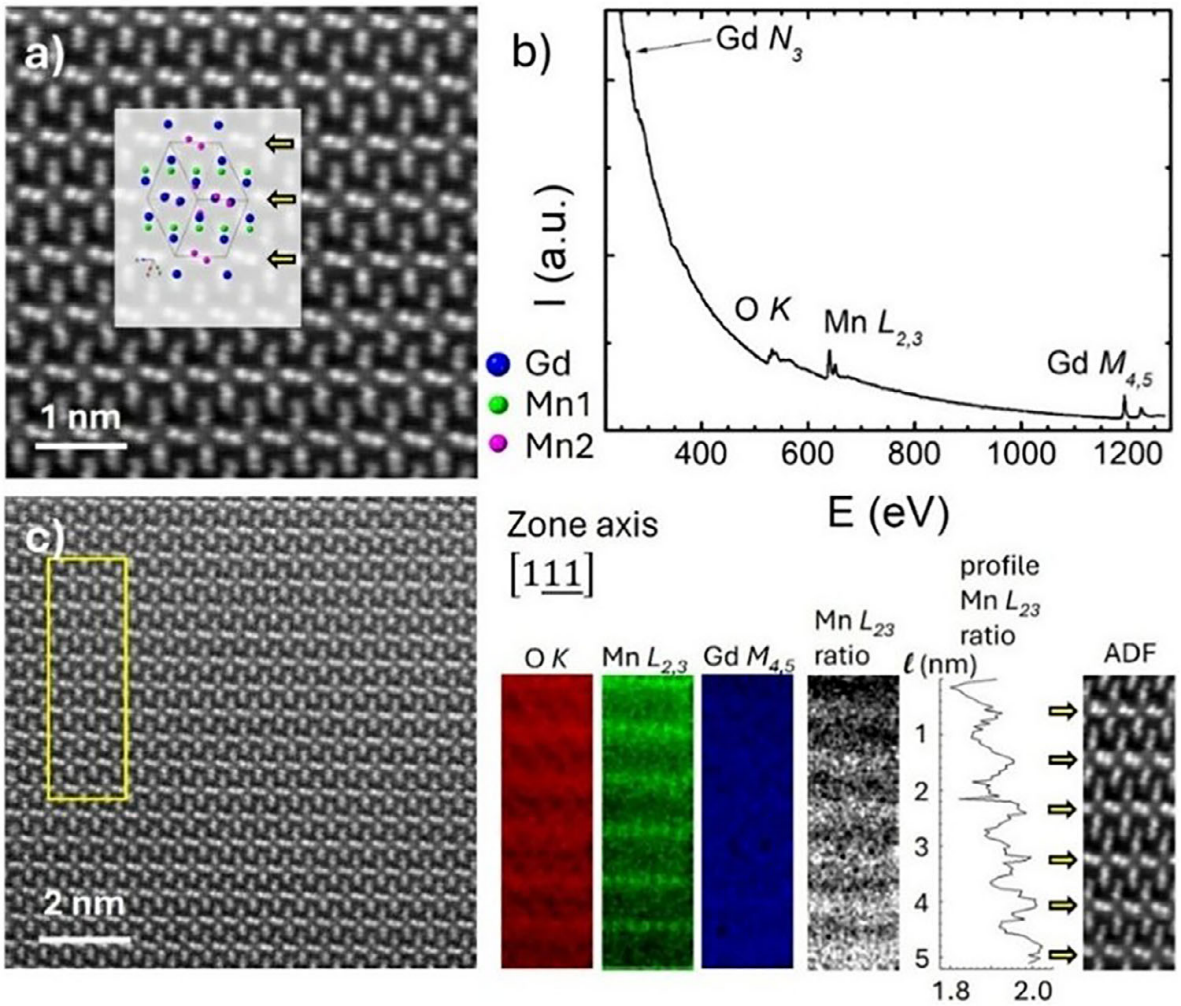
(a) atomic resolution HAADF image of GdMn_2_O_5_ crystal oriented along the [1−1−1] zone axis. The inset shows the unit cell model along this direction: blue spheres for Gd, green spheres for Mn_1_ (octahedral site), and pink spheres for Mn_2_ (pyramidal site). Oxygen atoms are not visible in the HAADF image and correspondingly not plotted in the model. The yellow arrows point to layers containing only Mn2 ions. (b) EEL spectrum of GdMn_2_O_5_ with edges used for the quantification: O *K*, Mn *L_2,3_* and Gd *M_4,5_*. (c) The crystal region used for EEL spectroscopy (yellow rectangle) and the corresponding elemental maps. On the right, the map of Mn *L_2,3_* ratio and its profile along the vertical axis is shown. Finally, the simultaneous HAADF image is shown with the same yellow arrows pointing to Mn_2_ layers where Mn *L_2,3_* ratio is higher than in Mn_1_ layers.

Figure 2 shows the atomic resolution STEM images and spectroscopy of the GdMn_2_O_5_ crystal. In these HAADF images (a,c) the crystal is oriented along the [1−1−1] zone axis and the incrusted model enable us to identify heavy Gd atoms (blue spheres) and observe separate layers of Mn_1_ ions (green spheres) and Mn_2_ (pink spheres). The light oxygen atoms are not visible in HAADF images and consequently oxygen atoms are also omitted from the crystal model shown here. The EELS quantification is made based on the O *K*, Mn *L_2,3_* and Gd *M_4,5_* edges, shown in panel b). The elemental maps are shown in panel c. On the right end of these maps, we have plotted the Mn *L_2,3_* ratio and its vertical profile, together with the simultaneous ADF image. The Mn *L_2,3_* edge, equally as *L_2,3_* edges of other transition metals (Ti–Zn) are known to bear the signature of the metals electronic state. The intensity ratio of *L_3_* and *L_2_* peaks (*L_3_* peak is at lower energy than *L_2_* peak) depends on the electron occupation of metals 3*d* orbitals [24], and an extensive study on manganite's has shown that the Mn *L_2,3_* ratio decreases when manganese ions are in higher oxidation states [25]. While the absolute value of Mn *L_2,3_* ratio depends on the quantification method, the tendency of lower Mn *L_2,3_* ratio for higher Mn oxidation state is universally observed and accepted. The Mn *L_2,3_* ratio map together with its profile indicates that Mn_2_ ions (pyramidal coordination, indicated by yellow arrows) are less oxidized than Mn_1_ ions in octahedral coordination. This profile clearly indicates that charge order of Mn ions is present in GdMn_2_O_5_. The *L_2,3_* ratio changes between Mn_1_ and Mn_2_ ions is rather small (according to our calibration, this corresponds to only 0.12 electrons between two types of manganese ions) but this can be easily understood by the scarce distance between two types of manganese ions and, consequently, mixing of their spectroscopic signals.

### 2.3 X‐Ray Absorption Spectroscopy at Low Temperatures

**FIGURE 3:**
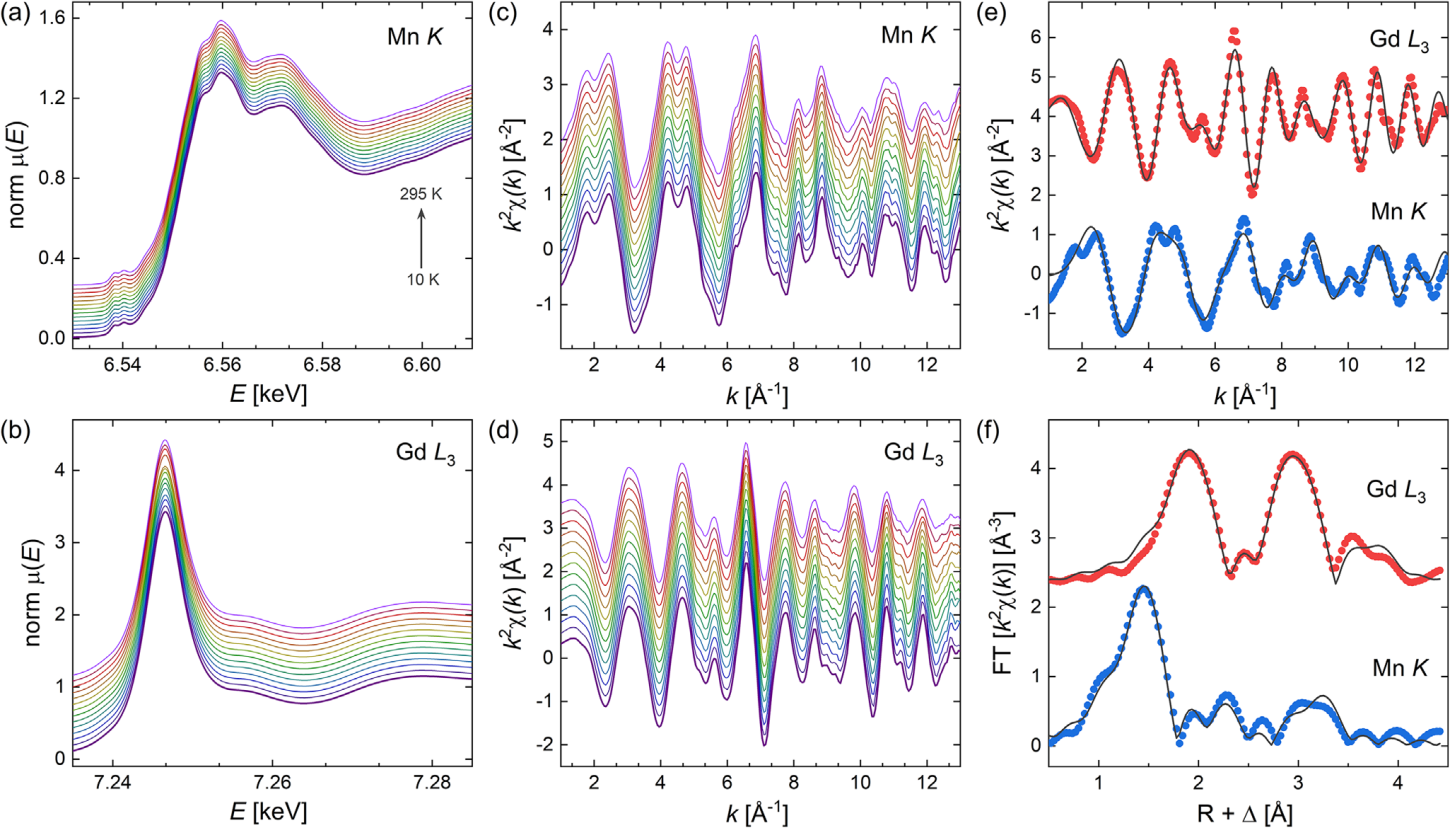
Normalized XANES spectra recorded at Mn *K*‐edge (a) and Gd *L*_3_‐edge (b) as a function of temperature from 10 to 295 K (vertically shifted). Corresponding *k* ^2^‐weighted EXAFS oscillations *k* ^2^·χ(*k*) at the Mn *K*‐edge (c) and Gd *L*_3_‐edge (d), showing the evolution of the local atomic structure around the Mn and Gd sites, respectively, across the full temperature range. (e) *k* ^2^‐weighted EXAFS oscillations *k* ^2^·χ(*k*) at Gd *L*_3_‐edge (red) and Mn *K*‐edge (blue) recorded at 10 K, with experimental data shown as open symbols and best fits as black solid lines. (f) Corresponding Fourier transform magnitudes FT[*k* ^2^·χ(*k*)] (not phase‐shift corrected), elucidating the resolved coordination shells around Gd and Mn absorbing sites at 10 K. The excellent agreement between experimental data and fitted curves at both edges confirms the reliability of the two‐sublattice structural model, resolving the Mn–O, Mn···Mn_1_/M_2_, and Mn···Gd shells at Mn *K*‐edge, and the split Gd–O_1_/O_2_, Gd···Mn, and Gd···Gd shells at Gd *L*_3_‐edge.

Temperature‐dependent X‐ray absorption spectroscopy measurements were performed at the Mn *K*‐ and Gd *L*_3_‐edges of GdMn_2_O_5_ between 10 and 295 K to probe the local atomic and electronic structure across the entire investigated temperature range. Figure 3a,b present the normalized XANES spectra at Mn *K*‐ and Gd *L*_3_‐edges, respectively, while the corresponding EXAFS oscillations, *k* ^2^·χ(*k*) are shown in Figure 3c,d. At both absorption edges, the XANES line shapes remain remarkably stable upon cooling and warming, exhibiting only subtle temperature‐induced variations in peak intensity and energy position. The first‐derivative spectra confirm the absence of detectable edge shifts within the experimental resolution (not represented here), indicating that the average oxidation states of Mn and Gd remain unchanged throughout the investigated temperature interval. The Mn *K*‐edge (1*s* → 4*p* transition) and Gd *L*_3_‐edge (2*p*_3/2_ → 5*d* transition) probe the unoccupied *p* and *d* states of the respective absorbing atoms, providing direct site‐selective sensitivity to changes in oxidation state, local coordination geometry, and orbital hybridization at Mn and Gd sites. Consistent with this observation, the EXAFS oscillations display a progressive enhancement of amplitude at low temperature, reflecting the reduction of thermal disorder, while preserving their overall phase and frequency characteristics. No abrupt modifications, peak splitting, or anomalous redistribution of spectral weight are observed in either reciprocal or real space representations. These results indicate that the local environments surrounding both Mn and Gd evolve smoothly with temperature and do not exhibit signatures of a structural phase transition between 10 and 295 K. The high quality of the EXAFS signal and the systematic thermal evolution of the oscillation amplitudes provide a robust basis for quantitative modeling of the local structure, which is presented in the following paragraphs through detailed EXAFS refinements and analysis of the temperature dependence of the mean‐square relative displacements.

The local atomic structure around the Mn and Gd absorbing atomic sites in GdMn_2_O_5_ was quantitatively investigated through EXAFS analysis at Mn *K*‐ and Gd *L*_3_‐edges, respectively. Structural parameters extracted from EXAFS best fitting at 10 K are listed in Table S2. At the Mn *K*‐edge, the best‐fit structural model comprises four single‐scattering paths. The first coordination shell is defined by the Mn–O path with a mean distance of 1.906 Å and a coordination number of 5.5, consistent with the coexistence of [Mn^3+^O_5_] square pyramids and [Mn^4+^O_6_] octahedra in the GdMn_2_O_5_ structure, whose average yields a non‐integer effective coordination intermediate between five and six. The relatively low bond‐length variance (σ^2^ = 1.99 × 10^−3^ Å^2^) at this shell indicates a well‐ordered and structurally rigid first coordination environment around Mn. The second shell, attributed to Mn···Mn_1_ correlations at 2.888 Å with *N* = 3, corresponds to edge‐sharing Mn polyhedra and is characterized by a markedly larger variance (σ^2^ = 11.31 × 10^−3^ Å^2^), reflecting the structural frustration and disorder inherent to the zigzag Mn sublattice topology. The third shell at 3.335 Å, assigned to Mn···Gd correlations (*N* = 5), captures the intersite Mn–Gd coupling with a moderate bond‐length variance (σ^2^ = 4.54 × 10^−3^ Å^2^), providing direct local structural evidence of the spatial proximity and potential exchange interaction between the two magnetic sublattices. The fourth shell at 3.496 Å, attributed to more distant Mn···Mn_2_ pairs (*N* = 4), presents the largest variance of the model (σ^2^ = 16.81 × 10^−3^ Å^2^), indicative of significant bond‐length dispersion associated with the geometrically frustrated corner‐sharing connectivity of Mn polyhedra at this range.

At the Gd *L*_3_‐edge, the structural model reveals a more ordered local environment around the Gd^3+^ site. The first coordination shell is split into two distinct Gd–O contributions: Gd–O_1_ at 2.357 Å (*N* = 5) and Gd–O_2_ at 2.462 Å (*N* = 3), both sharing the same bond‐length variance (σ^2^ = 2.70×10^−3^ Å^2^), consistent with the distorted square antiprismatic coordination environment of Gd^3+^ in the GdMn_2_O_5_ structure, where oxygen ligands occupy two crystallographically inequivalent positions at slightly different distances. The splitting of the Gd–O shell into two sub‐shells with a separation of ∼0.10 Å provides direct spectroscopic evidence of the anisotropic nature of the Gd^3+^ coordination polyhedron. The third shell at 3.333 Å, attributed to Gd···Mn correlations (*N* = 6), is remarkably close to the Mn···Gd distance extracted from Mn *K*‐edge fit (3.335 Å), demonstrating excellent self‐consistency of the structural model across both edges and confirming the robustness of the refined Mn–Gd intersite distance. The low variance of this path (σ^2^ = 2.50 × 10^−3^ Å^2^) further suggests a relatively rigid Gd–Mn structural framework, potentially reflecting the moderate but non‐negligible Gd–Mn exchange coupling responsible for the pressure‐dependent enhancement of *T*_Gd_ reported in this work. Finally, the most distant shell at 3.893 Å, assigned to Gd···Gd correlations (*N* = 2), exhibits the smallest variance of the entire model (σ^2^ = 0.72 × 10^−3^ Å^2^), indicating a highly ordered and well‐defined Gd–Gd interatomic distance, consistent with the crystallographic regularity of the Gd sublattice within the *Pbam* orthorhombic framework.

The EXAFS results at both edges paint a coherent picture of the local structure of GdMn_2_O_5_: a disordered and frustrated Mn sublattice, as evidenced by the large bond‐length variances of the Mn–Mn shells, coexisting with a more ordered and rigid Gd sublattice. The self‐consistent Mn···Gd and Gd···Mn distances retrieved independently from two edges establish the reliability of the structural model and highlight the intimate spatial coupling between the two magnetic sublattices, which underpins the sublattice‐selective pressure response discussed in this work.

### 2.4 Magnetization and Specific Heat Studies

**FIGURE 4:**
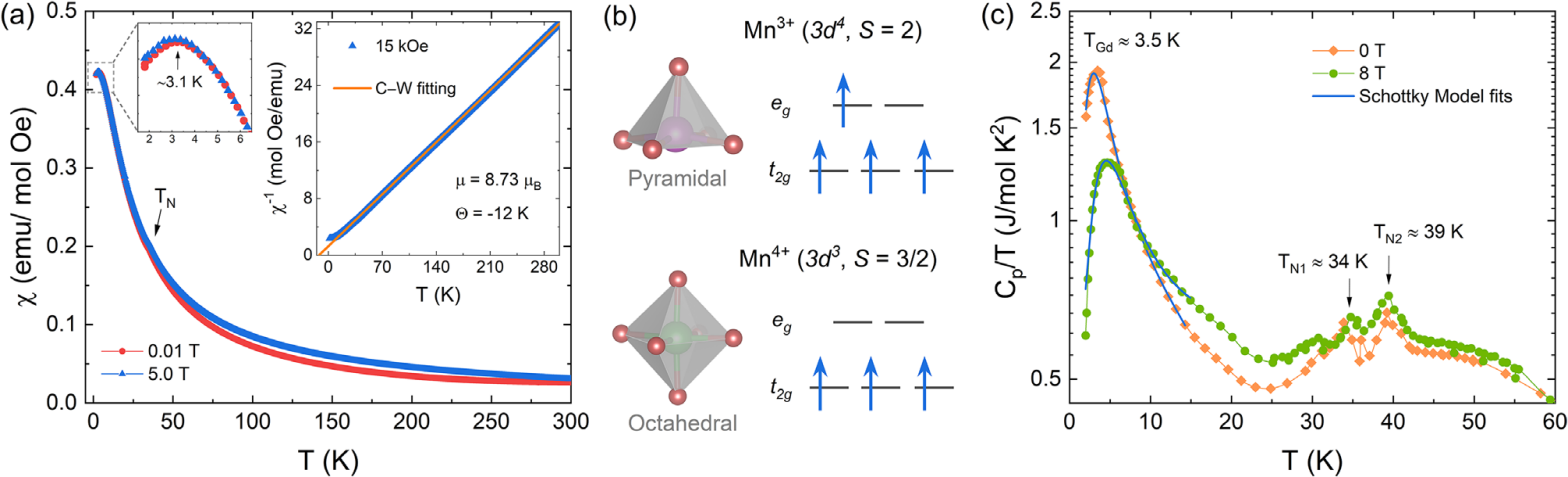
(a) Temperature dependence of the magnetic susceptibility *χ*(*T*) at FC protocol for GdMn_2_O_5_ under the magnetic fields of 0.01 and 5 T. Insets display the zoom of *χ*(*T*) curves at low temperature (left) and inverse *χ*(*T*) fitted with C–W law (right). (b) *C_p_*/*T*(*T*) curves recorded under applied fields of 0 and 8 T. Blue solid lines are two‐level Schottky‐type fittings.

Figure 4a presents the temperature‐dependent magnetic susceptibility *χ*(*T*) curves of GdMn_2_O_5_ measured under external fields of 0.01 and 5 T in the field cooling (FC) protocol. The *χ*(*T*) curves suggest an antiferromagnetic (AFM) behavior at low temperatures with transition to a paramagnetic (PM) state at Néel temperature of *T_N_* ≈ 34 K (by the first derivative *dχ/dT*), whereas the anomaly at *T_Gd_* ≈ 3.1 K (inset left) corresponds to the AFM ordering of Gd^3+^ ions. A secondary feature at *T_SR_* ≈ 34 K is attributed to a spin‐reorientation transition of the Mn sublattice. Evidence of AFM‐like short‐range interactions is provided by the unsaturated isotherms magnetization *M*(*H*) at low temperatures, whereas the PM behavior is confirmed at 300 K (see Figure S1). As shown in inset (right) of Figure 4a, above *T_N_*, the inverse susceptibility follows the linearity of Curie–Weiss (C–W) law 𝜒^−1^ = [C/(T − 𝜃_P_)]^−1^, wherein C and 𝜃_P_ are the Curie constant and paramagnetic C–W temperature, respectively. The C–W fitting result in negative 𝜃_P_ = ‐12 K, indicating the AFM interactions at the ground state. The deduced value of effective magnetic moment is found to be *µ_eff_* = 8.73 µ_B_/f.u. using $\mu_{eff}=\sqrt{8C}$, which agrees reasonably with the theoretical expectation of 10.1 µ_B_ given by $\mu_{theo}=\sqrt{{\left(\mu_{Gd^{3+}}\right)}^{2}+{\left(\mu_{Mn^{3+}}\right)}^{2}+{\left(\mu_{Mn^{4+}}\right)}^{2}}$, considering the combination of magnetic moments of Gd^3+^ (*J* = 7/2, *µ* = 7.94 µ_B_), and Mn^3+^ (*S* = 2, *µ* = 4.9 µ_B_) and Mn^4+^ (*S* = 3/2, *µ* = 3.87 µ_B_) in the high‐spin (HS) state (as illustrated in Figure 4b). This reduced *µ_eff_* arises from the non‐collinear AFM order and geometric frustration inherent to the GdMn_2_O_5_ structure, where the Gd^3+^, Mn^3+^, and Mn^4+^ spins are not aligned parallel but instead adopt a complex, partially cancelled configuration [26]. The weak exchange striction between 4*f*–3*d* moments, together with the dominant Mn^3+^–Mn^4+^ interactions, does not compensate for the frustration‐induced moment reduction, ultimately yielding a lower net magnetic moment.

To further investigate the temperature‐induced transitions, we performed specific heat (*C_p_*) measurements as a function of temperature under applied fields of 0 and 8 T (Figure 4c). Notably, the *C_p_*/*T*(*T*) curves clearly display three distinct anomalies, each marked by a sharp peak. The peak at *T_Gd_* ≈ 3.5 K corresponds to the typical AFM ordering of the Gd^3+^ magnetic moments [16]. The peak at *T_N_* ≈ 39 K arises from the AFM ordering of the Mn^3+^/Mn^4+^ ions, whereas the peak at *T_SR_* ≈ 34 K signals a spin reorientation from an incommensurate to a commensurate magnetic state, in agreement with previous reports [12, 27]. Particularly, upon applying an 8 T field, this peak weakens and shifts slightly to a higher temperature, indicating a field‐induced suppression of spin fluctuations and a stabilization of magnetic correlations. The low‐temperature *C_p_* (*T*)/*T* curves were further analyzed using a two‐level Schottky‐type contribution with non‐degenerate levels, combined with a smooth low‐temperature magnetic background [14]. The fits yield effective Gd^3+^ magnetic splitting of Δ/*k_B_* ∼ 9.9 K at 0 T and ∼ 14.7 K at 8 T. The field‐induced increase is quantitatively consistent with the Zeeman scale expected for a spin‐only Gd^3+^ ion, since $\sqrt{\frac{\Delta^{2}\left(8T\right)-\Delta^{2}\left(0T\right)}{k_{B}}}$ ≈ 10.87 K is close to $\frac{g_{J}\mu_{B}H}{k_{B}}$ ≈ 10.8 K for μ_0_ *H* = 8 T. In addition, the 0 T specific heat displays a small residual peak/feature near 4 K that is not reproduced by the Schottky contribution, consistent with the Gd^3+^ magnetic‐ordering anomaly observed in the magnetic susceptibility. This feature is no longer resolved at 8 T, where the Schottky term plus a reduced linear background describes the low‐temperature data. Since GdMn_2_O_5_ is insulating, the linear term is unlikely to be electronic and is more naturally associated with low‐energy magnetic correlations or excitations; its 30% reduction under field suggests partial suppression or polarization of Gd‐related magnetic degrees of freedom.

### 2.5 Magnetocaloric Performance

**FIGURE 5:**
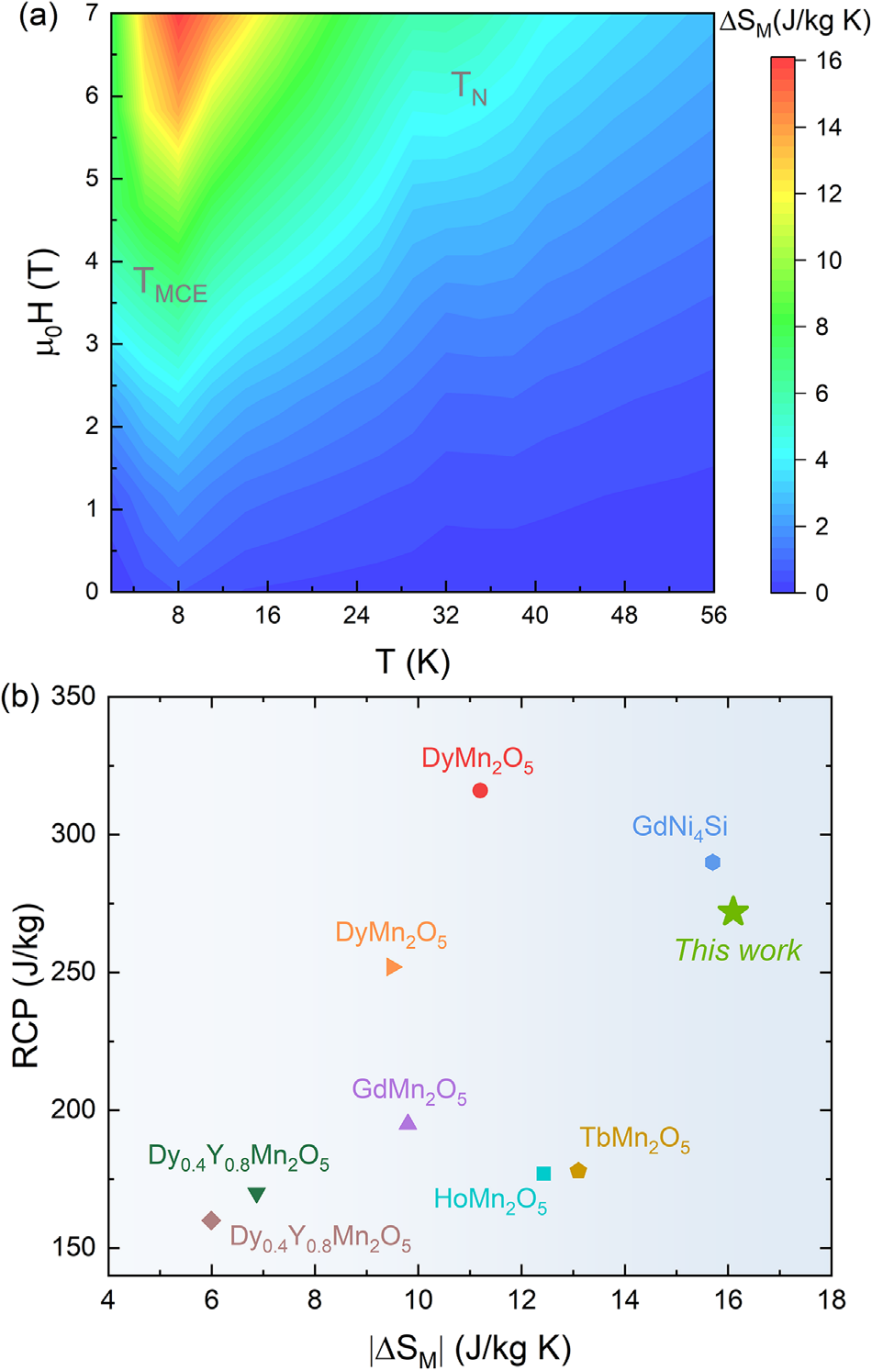
(a) Contour plot of Δ*S_M_* as a function of temperature and external magnetic field for GdMn_2_O_5_. (b) Comparing Δ*S_M_* and *RCP* values with similar magnetocaloric materials operating at cryogenic temperatures below 25 K.

The magnetocaloric effect (MCE), quantified by the magnetic entropy change (Δ*S_M_*), was determined from isothermal magnetization *M*(*H*) curves measured in the 2–62 K temperature range (shown in Figure S2a). This was achieved by numerical integration of Maxwell's thermodynamic relation [21], as given by:

(1) $$\begin{array}{ccc} \Delta S_{M}\left(T,H\right) & = & \int_{0}^{H}{\left(\frac{\partial M}{\partial T}\right)}_{H}\;dH\to \Delta S_{M}\left(T,H_{0}\right)\\ & = & \sum_{i}\frac{M_{i+1}-M_{i}}{T_{i+1}-T_{i}}\;\Delta H_{i} \end{array}$$

where, *M_i_* and *M_i+1_* are the magnetization obtained at temperatures *T_i_* and *T_i+1_*, under a magnetic field *H_i_*, respectively. Firstly, we transformed the *M*(*H*) curves into Arrott‐plots (*H*/*M* vs. *M* ^2^) using the Banerjee criterion [28] (see Figure S2b). All curves exhibit a positive slope, indicating that GdMn_2_O_5_ undergoes a second‐order magnetic phase transition (SOPT). This behavior inherently minimizes thermal and field hysteresis, which is advantageous for achieving stable and reversible magnetocaloric performance in practical refrigeration cycles. Figure 5a displays the ‒Δ*S_M_* (*T*) curves for GdMn_2_O_5_ under external magnetic field changes from 0–1 to 0–7 T. The curves exhibit a conventional MCE, characterized by well‐defined positive peaks around *T_MCE_* ≈ 8 K (at 0–7 T), which can be attributed to the partially induced magnetic moment of the Gd^3+^ ions, and another, less intense one, around *T_N_* ≈ 39 K. The $\Delta S_{M}^{max}$ follows the power law $\Delta S_{M}^{max}\approx {\left(\mu_{0}H\right)}^{n}$ as demonstrated in Figure S3, yielding an exponent *n* = 1.48 that is greater than those of mean‐field ferromagnets (*n* = 0.67), which is another evidence for the AFM interactions. At lower field changes, the −Δ*S_M_* reaches ∼1.9 J/kg K at 0–2 T, and ∼9.6 J/kg K at 0–5 T. Notably, the maximum value of $\Delta S_{M}^{max}$ ≈ 16.1 J/kg K at 0–7 T, which exceeds the values previously reported for GdMn_2_O_5_ (9.8 J/kg K) [12], and DyMn_2_O_5_ (11.2 J/kg K) [11]. Moreover, our value is also comparable to those reported for other *R*Mn_2_X_2_‐related compounds. For instance, TmMn_2_Si_2_ exhibits a large reversible MCE of ∼24.9 J/kg K at 5.5 K [29], whereas DyMn_2_Ge_2_ single crystals show ∼18.8 J/kg K at 39 K under 0–7 T [30].

Furthermore, the adiabatic temperature change (Δ*T_ad_* ​) was determined from the heat‐capacity curves collected at 0 and 8 T by calculating the field‐dependent entropy through numerical integration of *C_p_*/*T* and applying the constant‐entropy condition, Δ*T_ad_* (*H*,*T*) =  *T*(*S*, *H_f_*) − *T*(*S*, *H_i_*) [31, 32]. The resulting Δ*T_ad_* ​(*T*) reaches a maximum value of ∼2.6 K under 0–8 T near 12 K (Figure S4). This value is comparable to those reported for recently developed Gd‐based cryogenic magnetocaloric materials, such as Gd_9.33_[SiO_4_]_6_O_2_ [33] and Gd_2_MnZnO_6_ [34].

Lastly, we evaluated the effectiveness of magnetic refrigeration through relative cooling power (*RCP*) parameter [15], defined by:

(2) $$RCP=\left|\Delta S_{M}^{max}\right|\;\times \;\delta T_{FWHM}$$

where, δ*T_FWHM_* is full width at half maximum of ‐Δ*S_M_* (*T*) curves. The *RCP* magnetic field‐dependent together with their best fit using power‐law *RCP* ≈ μ_0_ *H^m^* yielded *m* = 1.53 (Figure S3), which reveals a greater field response similarly to Δ*S_M_*. This result indicates that the *RCP* can be significantly enhanced by increasing the applied magnetic field. Notably, the maximum value reaches *RCP^max^* ≈ 272 J/kg (at *µ_0_ H* = 0–7 T) which is comparable or exceeds the values reported for Gd‐based compounds like Gd_5_Ge_2_Si_2_ (240 J/kg) [35] and GdNi_4_Si (322 J/kg) [36].

To contextualize the MCE performance of GdMn_2_O_5_, we compared the Δ*S_M_* and *RCP* values with similar magnetocaloric materials [10, 11, 12, 13, 36] operating at cryogenic temperatures below 25 K, as presented in Figure 5b. Remarkably, the results for our GdMn_2_O_5_ significantly surpasses those of related *R*Mn_2_O_5_ compounds. This improvement can be attributed to its distinctive structural and magnetic properties. These materials adopt an orthorhombic *Pbam* structure composed of Mn^4+^O_6_ octahedral and Mn^3+^O_5_ pyramidal units, where the magnetic ordering is governed by Mn^3+^/Mn^4+^ networks, while the rare‐earth sublattice contributes to the dominant magnetic entropy change. Among the rare‐earth ions, Gd^3+^ is unique due to its *J* = 7/2 (*L* = 0) ground state, which eliminates crystal electric field anisotropy. As a result, the full spin entropy is released sharply at the ordering transition. Particularly, HoMn_2_O_5_ and DyMn_2_O_5_ exhibit lower peak Δ*S_M_* (∼12.4 and ∼9.5 J/kg K, respectively) [10, 13], as the anisotropic Dy^3+^ and Ho^3+^ experience crystal electric field level splitting, which broadens the magnetic transition, like to SOPT. Moreover, Y‐doping DyMn_2_O_5_ further dilutes the magnetic sublattice, reducing the MCE performance. Interestingly, our achieved *RCP* of 272 J/kg indicates a suitable operational cryogenic temperature, particularly when compared to non‐manganite materials such as GdNi_4_Si [36], which exhibits a comparable entropy change of 15.7 J/kg K, but a significantly lower *RCP*. Lastly, a direct comparison with the same GdMn_2_O_5_ compound reported by Mansouri et al. [12], which exhibits a Δ*S_M_* of 9.8 J/kg K, reveals that our sample demonstrates a substantially improved MCE performance of 16.1 J/kg K. This enhancement is attributed to the Gd^3+^ ground state (*J* = 7/2), which eliminates crystal electric field anisotropy, enabling a large field‐induced entropy change at the ordering transition. The measured value of 16.1 J/kg K corresponds to approximately 33% of the theoretical full Gd^3+^ spin entropy (R *ln* 8 = 49.7 J/kg K), which is consistent with the presence of competing AFM interactions in the frustrated Mn sublattice that suppress complete polarization of the Gd^3+^ sublattice at 7 T. Collectively, GdMn_2_O_5_ belongs to a family of oxide‐based magnetocaloric materials derived from relatively inexpensive and non‐toxic elements, making it a promising candidate for practical cryogenic magnetocaloric applications.

## 3 Discussion

### 3.1 Temperature‐Dependent Crystalline Structure

**FIGURE 6:**
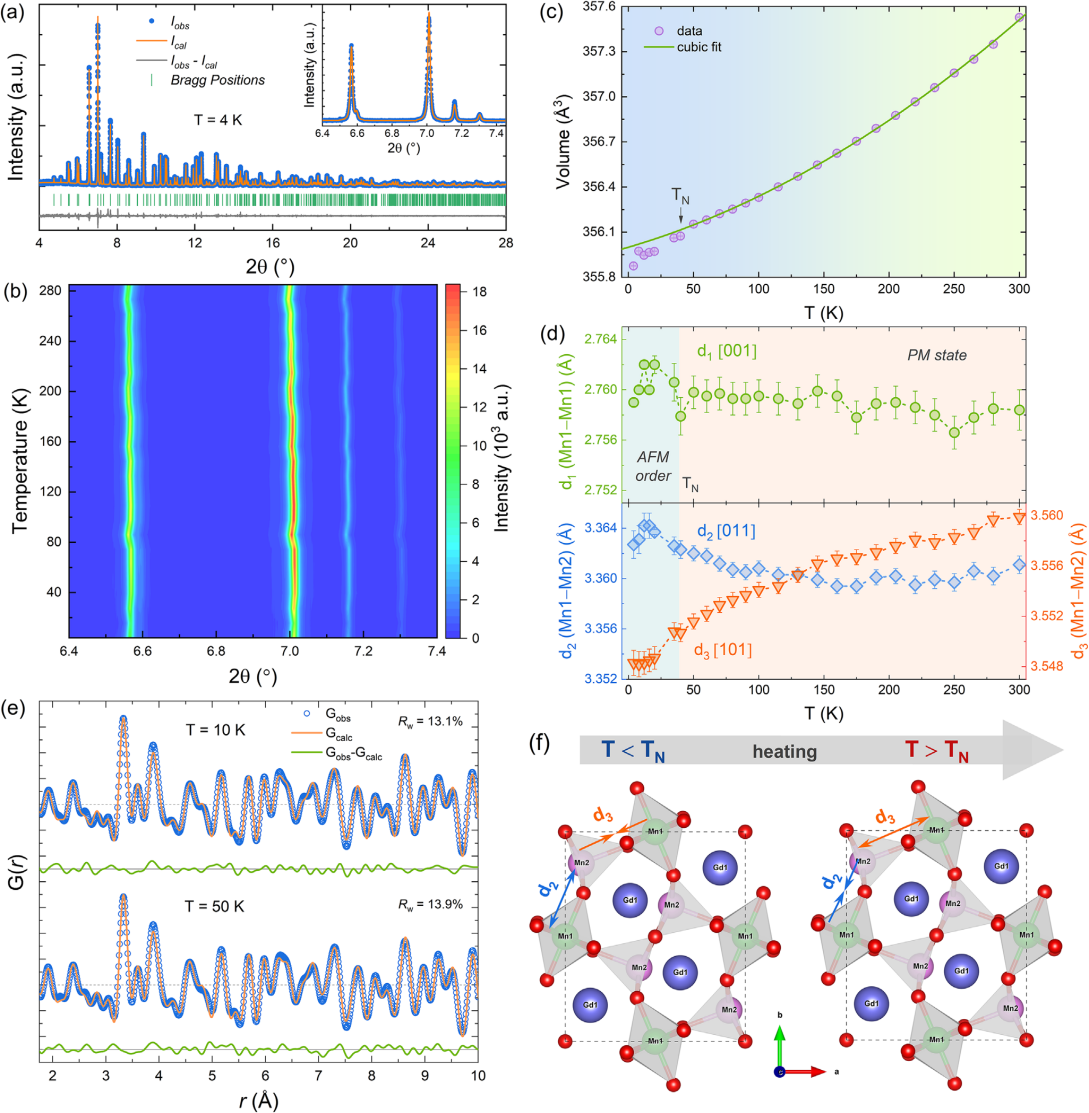
(a) Rietveld plot from SXRD data at 4 K. (b) Plot showing the intensity of SXRD peaks (color scale) as a function of temperature and angle (2θ). (c) Thermal expansion of the unit‐cell volume *V*(*T*), where the green line denotes a cubic function fit. (d) Temperature‐dependent neighboring Mn–interatomic distances along [001], [011], and [101] directions. (e) X‐ray PDF data and corresponding fits at 10 and 50 K, respectively. (f) Schematic representation of the nearest‐neighbor Mn_1_–Mn_2_ distances below and above *T_N_*, respectively.

To investigate the crystalline structure evolution of GdMn_2_O_5_ with temperature, we collected sequential SXRD patterns from 4 up to 280 K. Notably, the absence of peak splitting or new peak emergence throughout the investigated temperature range excludes a global structural phase transition down to 4 K (see Figure 6a,b). The temperature‐dependent unit cell volume (*V*) obtained from the Rietveld refinement analysis is shown in Figure 6c. The volumetric coefficient of thermal expansion, calculated by α_*V*_ = Δ*V*/*V*_0_ × Δ*T* (where *V_0_* is the initial volume and Δ*V* is the volume change corresponding to the temperature change Δ*T*), was found to be 1.56(2) ×10^−5^ K^−1^ [Δ*V* = 1.65(3) Å^3^] in the 4–300 K temperature range, suggesting that GdMn_2_O_5_ undergoes a reasonable crystalline thermal expansion.

Interestingly, we observed a negative thermal expansion in the *b*‐lattice parameter, in contrast to the normal behavior of the *a*‐ and *c*‐axes (see Figure S5), which correlates with the temperature‐dependent evolution of nearest‐neighbor Mn–Mn distances, as presented in Figure 6d. Particularly, *d*_2_ (Mn_1_–Mn_2_) distance along the [011] direction exhibits a clear contraction upon heating, which is fully consistent with the observed reduction of the *b*‐lattice parameter. This behavior strongly indicates a preferential strengthening of the Mn_1_–O–Mn_2_ super‐exchange interactions along this crystallographic direction. Near *T_N_*, anomalies are observed in both volume and Mn–interatomic distances (see Figure 6c,d), exhibiting a clear discontinuity or change in slope at the same temperature. These structural anomalies are directly associated with the magnetoelastic coupling intrinsic to the antiferromagnetic‐paramagnetic (AFM‐PM) transition, demonstrated earlier in magnetic analysis. As the system crosses *T_N_*, the onset of long‐range antiferromagnetic order drives a magnetoelastic effect, primarily through exchange striction, a mechanism whereby the magnetic exchange interactions become strongly coupled to the lattice degrees of freedom. The reorientation and ordering of magnetic moments at the transition generate internal lattice strains that distort the local crystal environment, particularly affecting the bond lengths between the two distinct Mn sites.

To examine the local structure of GdMn_2_O_5_ around *T*_*N*_, we carried out X‐ray atomic pair distribution function (PDF) analysis of synchrotron total scattering (TS) data collected at 10 and 50 K (Figure 6e). The local structure could be accurately modelled using the GdMn_2_O_5_ structure in the *Pbam* space group for both temperatures, yielding an excellent fit to the experimental data, consistent with our SXRD results. Moreover, the absence of additional split peaks or reduced symmetry in the PDF at both temperatures confirms that the local structure remains consistent with the average orthorhombic space group. The *d*_2_ distance determined by PDF analysis at 10 K (*T* < *T_N_*) is clearly found to expand along the [011] direction temperature cooling (Figure 6f). This expansion correlates directly with the increase in the *b*‐lattice parameter upon cooling, from the average SXRD refinement. However, the *d*_1_ and *d*_3_ distances along the [001] and [101] directions, respectively, exhibit markedly different temperature‐dependent behaviors, reflecting the anisotropic nature of the magnetoelastic coupling.

At 50 K, the same Mn_1_–Mn_2_ pairs show a slight elongation along the [101] direction (see Figure 6f) but contract along [011], indicating the onset of thermal fluctuations and the partial release of exchange‐striction‐induced lattice strain as long‐range magnetic order is lost. Indeed, the anisotropic thermal expansion observed in the Mn–Mn bond distances provide direct evidence that magnetic frustration drives strong magnetoelastic coupling in GdMn_2_O_5_. The AFM frustrated exchange interactions along the zig‐zag chains suppresses conventional collinear order, favoring a non‐collinear spin arrangement. This frustrated spin system is highly susceptible to lattice distortions via magnetoelastic coupling, where changes in the Mn_1_–Mn_2_ bond lengths alter the exchange energies and vice versa. Upon cooling across *T_N_*, the non‐collinear spin arrangement stabilizes and induces preferential expansion of Mn_1_–Mn_2_ distance along the [011] direction, a signature of exchange straining relieving frustration along that specific pathway. Conversely, above *T_N_*, thermal fluctuations partially release this lattice strain, leading to expansion along [101]. Thus, the strongly anisotropic thermal expansion of the lattice parameters reflects the underlying frustration, whereby the system selects a distortion pattern to minimize magnetic energy, evolving from frustrated short‐range order to a long‐range non‐collinear state. Remarkably, this magnetoelastic coupling reveals a precise correlation between structural anomalies and magnetic frustration, underscoring the intrinsic interdependence of spin order and crystal structure in GdMn_2_O_5_.

### 3.2 Local Structure and Electronic Features

**FIGURE 7:**
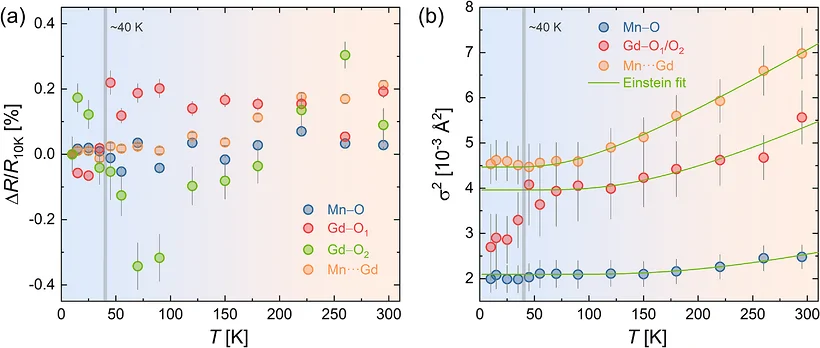
(a) Relative change in the EXAFS‐derived bond distance Δ*R*/*R*_10K_ for the different coordination shells (Mn–O, Gd–O_1_/O_2_, and Mn···Gd). (b) Bond‐length variance (σ^2^) obtained from Einstein‐model fits to the temperature‐dependent EXAFS data. Error bars represent the uncertainties from the EXAFS fitting procedure. The vertical dashed lines mark the reference *T_N_* temperature for GdMn_2_O_5_.

The temperature evolution of the local bond distances extracted from EXAFS analysis at the Mn *K*‐ and Gd *L*_3_‐edges provides compelling evidence for magnetoelastic coupling in GdMn_2_O_5_, as shown in Figure 7a. The relative bond‐length changes Δ*R*/ *R*_10K_ =  100 · [*R*(*T*) − *R*_10K_]/*R*_10K_, referenced to the 10 K values *R*_10K_, reveal markedly distinct thermal behaviors across the two sublattices and their interconnecting bonds, with anomalies that correlate directly with the onset of long‐range AFM order below *T_N_*. The Mn–O bond distance remains essentially temperature‐independent across the entire investigated range (10–295 K), with Δ*R*/*R*_10K_ confined within ± 0.05%, pointing to a structurally rigid and electronically stable first coordination shell around Mn. This rigidity is consistent with the strong covalent character of the Mn–O bonds within the [MnO_6_] octahedra and [MnO_5_] square pyramids and suggests that the Mn sublattice does not undergo significant local structural distortions upon magnetic ordering, in agreement with the absence of a global structural phase transition as confirmed by SXRD. In contrast, the Mn···Gd intersite distance exhibits a subtle but systematic contraction below *T_N_*, with Δ*R*/*R*_10K_ approaching zero from slightly negative values at the lowest temperatures, indicative of a weak but non‐negligible structural response of the Mn–Gd framework to the development of long‐range magnetic order.

The most striking evidence for magnetoelastic coupling is provided by the temperature evolution of the two inequivalent Gd–O bond distances. While the Gd–O_1_ distance follows a smooth and monotonic contraction of ∼0.20% upon cooling from 295 to 10 K, consistent with conventional anharmonic thermal contraction of the Gd coordination polyhedron, the Gd–O_2_ distance exhibits a pronounced and anomalous negative deviation below ∼100 K, with a well‐defined minimum of Δ*R*/*R*_10K_ ≈ −0.35% in the temperature interval 50–75 K, followed by a partial recovery toward lower temperatures. This anomalous contraction of the Gd–O_2_ bond sets in well above *T_N_*, suggesting that short‐range magnetic correlations within the Mn sublattice, which are known to develop significantly above the long‐range ordering temperature in frustrated magnets, already couple to the Gd local coordination geometry through Mn–Gd super‐exchange pathways. The subsequent partial recovery of *R*(Gd–O_2_) below *T_N_* may reflect a competing effect between the onset of Gd sublattice ordering and the Mn‐driven magnetoelastic distortion, a behavior reminiscent of sublattice‐selective magnetostriction reported in other rare‐earth manganites.

The temperature dependence of the bond‐length variance (σ^2^) extracted from EXAFS fitting at Mn *K*‐ and Gd *L*_3_‐edges provides compelling evidence for spin‐phonon coupling in GdMn_2_O_5_, as shown in Figure 7b. The dynamic part of each σ^2^ was modelled using the Einstein approximation:

(3) $$\sigma^{2}\left(T;\theta_{E},\sigma_{stat}\right)=\sigma_{stat}^{2}+\frac{\hbar^{2}}{2\mu k_{B}\theta_{E}}\coth\left(\frac{\theta_{E}}{2T}\right)$$

where *µ* is the reduced mass of the pair‐unit (Mn–O, Gd–O_1,2_, and Mn–Gd), θ_*E*_ is the Einstein temperature, and $\sigma_{stat}^{2}$ is the static (temperature‐independent) disorder [37, 38, 39, 40]. Following the approach established for spin‐phonon coupling in related perovskite oxides [41, 42, 43], the Einstein fits were performed exclusively for temperatures above 40–50 K (in the PM regime above *T_N_*) and subsequently extrapolated to lower temperatures to expose departures from purely anharmonic behavior at the onset of magnetic ordering. The three bond pairs exhibit markedly distinct thermal behaviors and Einstein temperatures, reflecting the fundamentally different character of each interaction. The Mn–O bond displays the highest Einstein temperature of θ_*E*_ ≈ 759 K.

This high stiffness is consistent with the strongly covalent character of the Mn–O bonds within the [MnO_6_] octahedra and [MnO_5_] square pyramids and is comparable to force constants reported for M–O bonds (M = Ni, Mn) in related transition‐metal oxides [44]. Crucially, the σ^2^(Mn–O) follows the Einstein model closely across the entire temperature range investigated, including below *T_N_*, indicating that the Mn–O bond vibrations are largely decoupled from the spin degrees of freedom. This result is consistent with the near temperature‐independence of the Mn–O bond distance discussed above and collectively indicates the Mn–O framework as a structurally and vibrationally rigid unit that does not participate directly in the magnetoelastic response of GdMn_2_O_5_. In contrast, the Gd–O bond yields a significantly lower Einstein temperature of θ_*E*_ ∼ 516 K, reflecting the more ionic and compliant character of the Gd–O coordination bonds relative to Mn–O, as expected from the larger ionic radius and lower charge density of Gd^3+^ compared to Mn^3+^/Mn^4+^. More importantly, the σ^2^(Gd–O) exhibits a pronounced departure from the Einstein fit below *T_N_*, with experimental values falling systematically below the extrapolated PM baseline. This suppression of bond‐length variance below the magnetic ordering temperature is the hallmark signature of spin‐phonon coupling as identified in the bond‐variance and has been previously reported in related magnetic oxides including PrNiO_3_ nickelate [43, 45] and DyFeO_3_ orthoferrite [46]. Physically, this anomaly arises because the development of long‐range AFM order below *T_N_* introduces an additional restoring force on the Gd–O bond vibrations through the modulation of the Gd–Mn exchange integral *J_ij_*, effectively stiffening the lattice and reducing the mean‐square relative displacement of the Gd–O pair below what is expected from purely thermal anharmonicity. This effect can be formally described by a perturbative correction to the Einstein temperature [43]:

(4) $${\left(\Delta \theta_{E}\right)}_{sp-ph}\approx -\frac{2\hbar^{2}}{\mu k_{B}^{2}\theta_{E}}\frac{\partial^{2}J_{ij}}{\partial u^{2}}{\left[\frac{M_{sub}\left(T\right)}{M_{0}}\right]}^{2}$$

where *M_sub_* (*T*) is the sublattice magnetization and $\frac{\partial^{2}J_{ij}}{\partial u^{2}}$ is the second derivative of the exchange integral with respect to the bond displacement *u*, in direct analogy with the spin‐phonon coupling model developed for LaMnO_3_ manganites [44] and later applied for PrNiO_3_ [43].

The most dramatic departure from Einstein behavior is observed for the Mn···Gd intersite pair, which yields the lowest Einstein temperature of θ_*E*_ ∼ 287 K, consistent with the weak and predominantly ionic character of this non‐bonding intersite interaction. The low stiffness of the Mn···Gd pair reflects the fact that this path does not correspond to a direct chemical bond but rather to the medium‐range structural coupling between the two magnetic sublattices through intervening oxygen bridges. Remarkably, σ^2^(Mn···Gd) exhibits the largest and most abrupt departure from the Einstein fit below *T_N_*, with experimental values rising significantly above the paramagnetic baseline upon cooling into the magnetically ordered state. This enhancement of bond‐length variance (as opposed to the suppression seen for Gd–O) indicates that the onset of AFM order in the Mn sublattice introduces additional dynamical disorder in the Mn–Gd intersite distance, most likely through the competing exchange interactions and geometric frustration of the Mn–O–Gd superexchange pathways. This behavior is qualitatively consistent with the anomalous contraction and recovery of the Gd–O_2_ bond distance observed in the Δ*R*/*R*_10K_ analysis, and reinforces the picture of a frustrated, magnetically soft Mn–Gd interface that acts as the primary structural transducer of the magnetoelastic coupling.

The hierarchy of Einstein temperatures [θ_*E*_(Mn–O) ≈ 759 K > θ_*E*_(Gd–O) ≈ 516 K > θ_*E*_(Mn···Gd) ≈ 287 K] reflects a clear gradient of bond stiffness from the rigid covalent Mn–O framework, through the intermediate ionic Gd–O coordination shell, to the compliant and frustrated Mn–Gd intersite coupling. The spin‐phonon anomalies, observed exclusively below *T*_*N*_ and most prominently for the Gd–O and Mn···Gd pairs, establish that the magnetoelastic coupling in GdMn_2_O_5_ is sublattice‐selective and bond‐specific: it is mediated predominantly through the Gd coordination environment and the Mn–Gd exchange pathway, while leaving the Mn–O covalent framework essentially unperturbed. These local structural and vibrational findings provide a microscopic basis for the macroscopic magnetocaloric response of GdMn_2_O_5_ and its pressure dependence, wherein the selective stiffening of the Mn–Gd exchange pathway under hydrostatic pressure directly modulates the temperature of maximum magnetocaloric entropy change.

### 3.3 Pressure Effects on the Magnetic and Magnetocaloric Behavior

**FIGURE 8:**
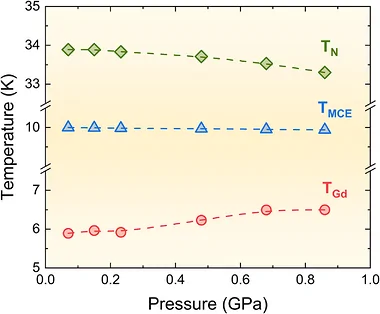
Pressure dependence of *T_N_*, *T_Gd_*, and *T_MCE_* in GdMn_2_O_5_. *T_N_* decreases with pressure, *T_Gd_* increases, while *T_MCE_* remains essentially constant. Dashed lines are guides to the eye.

Previous studies in the *R*Mn_2_O_5_ (*R* = rare earth) family have shown that hydrostatic pressure modulates magnetic interactions, inducing new ferroelectric phases and decoupling the rare‐earth moments [22, 23, 47]. However, the pressure response of the MCE in this family remains largely unexplored. Here, we investigate how hydrostatic pressure affects the magnetic transition temperatures of GdMn_2_O_5_, namely the Néel temperature of the Mn sublattice (*T_N_*), the Gd^3+^ ordering temperature (*T_Gd_*), and the MCE peak temperature (*T_MCE_*), aiming to understand the sublattice‐dependent pressure response of this multiferroic. Figure 8 shows the hydrostatic pressure dependence of *T_N_*, *T_Gd_*, and *T_MCE_*, determined from pressure‐dependent *χ*(*T*), *M*(*H*), and Δ*S_M_* (*T*) analyses (see Figures S6–S8). Remarkably, the three characteristic temperatures respond differently to applied pressure (see Figure 8). As pressure increases from 0.07 to 0.86 GPa, *T_N_* decreases from ∼33.9 to ∼33.3 K, similarly to other family materials like SmMn_2_O_5_ and NdMn_2_O_5_ [23]. This behavior is attributed to the weakening of the Mn–O–Mn super‐exchange interactions under lattice contraction. Hydrostatic pressure reduces the Mn–O bond lengths and modifies bond angles, which typically suppresses AFM ordering in manganese oxides.

In contrast, *T_Gd_* increases from ∼5.9 to ∼6.5 K over the same pressure range. This upward shift suggests that the effective molecular field acting on the Gd^3+^ moments from the ordered Mn sublattice may increase under compression, despite the decrease in *T_N_*. A possible explanation, consistent with previous reports on related *R*Mn_2_O_5_ compounds [22, 47], is that hydrostatic pressure could stabilize a new commensurate magnetic phase or modify the Mn spin configuration, potentially improving the magnetic coupling between the Mn and Gd sublattices. However, direct confirmation of such a pressure‐induced phase would require high‐pressure neutron or synchrotron diffraction studies. Moreover, the isotropic nature of the Gd^3+^ (^8^S_7/2_) ground state, which lacks crystal electric field anisotropy, allows it to respond sensitively to any enhancement in the local molecular field, resulting in a net increase of *T_Gd_* under pressure. On the other hand, the *T_MCE_* remains essentially constant under pressure, varying by less than 0.1 K across the entire range. Since this peak arises from the combined influence of the applied field and the molecular field from the Mn sublattice, its stability under pressure suggests that the temperature of maximum polarization efficiency is resilient to lattice compression.

The distinct pressure responses of *T_N_*, *T_Gd_*, and *T_MCE_* suggest a sublattice‐dependent tuning mechanism. The opposing pressure responses of *T_N_* and *T_Gd_* indicate that the Mn and Gd sublattices may respond independently to external stimuli, potentially offering a pressure‐based strategy for selectively tuning the magnetic ordering of the rare‐earth sublattice without significantly altering the MCE operating temperature. Further high‐pressure structural and magnetic studies would be required to fully establish this mechanism. Thus, GdMn_2_​O_5_​ emerges as a promising platform for sublattice‐selective pressure control in frustrated multiferroics, highlighting hydrostatic pressure as a key tool for tuning its magnetic properties.

## 4 Conclusion and Remarks

We have elucidated the structure–property relationships governing the magnetocaloric response of the multiferroic GdMn_2_O_5_ and its tunability under hydrostatic pressure. Synchrotron X‐ray diffraction and pair distribution function analyses confirm the orthorhombic *Pbam* structure over 4–300 K and reveal anisotropic thermal expansion driven by magnetoelastic coupling, without evidence of a structural phase transition. STEM‐EELS combined with site‐selective X‐ray spectroscopy reveals charge ordering between Mn sites and demonstrates that magnetoelasticity is governed by the compliant Gd–O environment and the frustrated Mn–Gd interface rather than the rigid Mn–O framework. Magnetic susceptibility and specific heat measurements identify successive magnetic ordering transitions at *T_N_* ≈ 39 K (Mn^3+^/Mn^4+^ ordering), *T_SR_* ≈ 34 K (spin‐reorientation), and *T_Gd_* ≈ 3.5 K (Gd^3+^ ordering). Under a field change of 0–7 T, GdMn_2_O_5_ exhibits a conventional MCE with a $\Delta S_{M}^{max}$ ≈ 16 J/kg K and *RCP* ≈ 272 J/kg (at ∼8 K), surpassing previously reported compounds and highlighting the favorable role of the isotropic Gd^3+^ ground state. Finally, hydrostatic pressure reveals a sublattice‐selective magnetic response: while *T_Gd_* increases under compression, consistent with strengthened Mn–Gd exchange interactions and stabilization of the commensurate magnetic phase, *T_MCE_* remains essentially unchanged. These results demonstrate that the Gd and Mn magnetic sublattices can be selectively manipulated by hydrostatic pressure, providing a strategy to tailor magnetic ordering independently of the magnetocaloric response. Overall, these findings highlight GdMn_2_O_5_ as a promising cryogenic magnetocaloric material and provide fundamental insight into the interplay between charge ordering, magnetoelastic coupling, and sublattice‐selective pressure responses in frustrated multiferroics.

## 5 Experimental Section

### 5.1 Synthesis

We have successfully synthesized a polycrystalline GdMn_2_O_5_ sample as a dark‐brown polycrystalline powder, starting from precursors previously synthesized by a citrate technique. Stoichiometric amounts of analytical grade Gd_2_O_3_ and Mn(NO_3_)_2_·4H_2_O were dissolved in citric acid. The citrate solution was slowly evaporated and decomposed at temperatures up to 600°C. All the organic materials were eliminated in a subsequent treatment at 800°C in air. A final treatment in air at 1050°C was required for achieving a pure specimen.

### 5.2 X‐Ray Diffraction and Analysis

The Synchrotron X‐ray diffraction (SXRD) data were collected using the high‐resolution powder diffractometer on beamline ID22 at the European Synchrotron Radiation Facility (ESRF) [48] (experiments MA‐5866 and HC‐6299). The SXRD patterns were collected at a wavelength of λ = 0.354 Å (∼35 keV) over the range 1−40° (2θ) in continuous scanning mode at 4–300 K range with a 5 min waiting time at each temperature step to guarantee isothermal condition. The high‐resolution setup was used at all times for the experiment, comprising a Dectris Eiger2×2M‐W CdTe pixel detector coupled with a 13‐channel Si(111) multi‐analyser stage. The samples were contained in 0.5 mm capillaries which were continuously rotating during the data acquisition. The final data were retrieved using the advanced processing implemented on ID22 [49]. Rietveld analysis of the SXRD data was performed using the *FullProf Suite* software package [50]. The crystal structure of GdMn_2_O_5_ was refined based on an orthorhombic structure defined in the *Pbam* space group (no. 55) [51]. The atomic occupancies were kept fixed to match the nominal GdMn_2_O_5_ composition, while atomic positions, scale factor, unit cell parameters, zero shift, and isotropic thermal parameters (*B*_iso_) were refined. The background was described by a linear interpolation between a set of background points with refinable intensity. The peak profiles were modelled using the Thompson–Cox–Hastings formulation of the pseudo‐Voigt function [52]. The PDFs were obtained using the *PDFgetX3* software [53]. To allow isolation of the scattering from the sample itself for PDF analysis, the scattering pattern from an empty 0.5 mm borosilicate capillary in the same instrumental configuration was subtracted. The real‐space structural refinements of the PDFs were carried out using the *PDFgui* software [54].

### 5.3 Transmission Electron Microscopy

Scanning transmission electron microscopy (STEM) and electron energy loss spectroscopy (EELS) are done in a JEOL ARM 200 electron microscope, equipped with an aberration corrector and Gatan Quantum EELS spectrometer. The microscope is operated at 200 kV.

### 5.4 X‐Ray Absorption Spectroscopy and Analysis

X‐ray absorption spectroscopy (XAS) measurements at the Gd *L*_3_‐edge (7.243 keV) and Mn *K*‐edge (6.539 keV) were carried out at the BM23 X‐ray absorption spectroscopy beamline [55] of the ESRF (Grenoble, France), using a fixed‐exit double‐crystal monochromator equipped with LN_2_‐cooled Si(111) crystals (experiment HC‐5654). Finely ground GdMn_2_O_5_ powder was diluted in cellulose and pressed into 6 mm pellets with mass ratios optimized to yield an edge jump of Δ*µx* ≈ 1.0 at each edge, minimizing thickness artifacts. Temperature‐dependent spectra from 10 to 295 K were collected in transmission geometry using a helium‐flow cryostat (± 0.1 K stability), with ionization chambers filled with N_2_/He and Ar gas mixtures; energy calibration was continuously monitored against Gd and Mn reference foils recorded simultaneously in a third downstream ionization chamber, ensuring absolute energy reproducibility better than 0.1 eV. Spectra were continuously acquired over the energy ranges 7.1–7.9 and 6.4–7.2 keV for the Gd *L*_3_‐ and Mn *K*‐edges, respectively, sampled at 1600 equally spaced points per scan, and subsequently rebinned to 0.2 eV steps in the XANES region and 0.02 Å^−1^ steps in the EXAFS region up to *k* = 14 Å^−1^ for both edges; multiple scans were averaged following careful energy alignment and glitch removal, with all data reduction carried out using the *ATHENA* software [56]. EXAFS analyses were performed in the framework of *ARTEMIS* software [56] within the *IFEFFIT* package.

### 5.5 Magnetic Studies

Magnetic properties were assessed utilizing a SQUID magnetometer (MPMS‐3) from Quantum Design (San Diego, USA) across temperatures ranging from 1.8 up to 300 K and magnetic fields up to 7 T. The heat capacity was measured in a PPMS with an adiabatic heat pulse method. For this, the sample pellets were cut with a diamond saw to fit into the sample‐holder dimensions.

## Author Contributions

**Javier Gainza**: investigation, validation, writing – review and editing, formal analysis, data curation. **João E. Rodrigues**: investigation, writing – original draft, methodology, validation, writing – review and editing, formal analysis, data curation. **Olivier Mathon**: validation, data curation, writing – review and editing. **Henrik L. Andersen**: investigation, writing – review and editing, formal analysis, validation. **Romualdo S. Silva**: conceptualization, investigation, writing – original draft, formal analysis, methodology, data curation, writing – review and editing. **Norbert M. Nemes**: investigation, writing – review and editing, validation, formal analysis. **José L. Martínez**: investigation, writing – review and editing, validation, formal analysis, data curation, funding acquisition, methodology. **Federico Serrano‐sánchez**: investigation, writing – review and editing, validation, data curation. **José A. Alonso**: funding acquisition, writing – review and editing, validation, formal analysis, data curation, supervision, project administration, methodology. **Neven Biskup**: investigation, writing – review and editing, validation, formal analysis, data curation.

## Funding

The present research project was partially funded by the Spanish Ministry for Science and Innovation (MCIN/AEI/10.13039/501100011033) for granting the projects: PID2021‐122477OB‐I00 and PID2025‐169160OB‐I00.

## Conflicts of Interest

The authors declare no conflicts of interest.

## Supporting information




**Supporting File**: advs77490‐sup‐0001‐SuppMat.docx.

## Data Availability

The data that support the findings of this study are available from the corresponding author upon reasonable request.
