# Supplementary material for: Sublattice‐Resolved Magnetoelastic Coupling and Opposing Pressure Responses in the Frustrated Magnetocaloric GdMn2O5
Source: Adv Sci (Weinh). 2026 Aug 31:e77490. Online ahead of print. doi: 10.1002/advs.77490 (PMC13528177; doi:10.1002/advs.77490)
Supplement: Supplementary file 1 [file ADVS-9999-e77490-s001.docx]

Supporting Information

Sublattice-Resolved Magnetoelastic Coupling and Opposing Pressure Responses in the Frustrated Magnetocaloric GdMn_2_O_5_

Romualdo S. Silva Jr.^1,*^, João E. Rodrigues^2,*^, Javier Gainza^2^, Henrik L. Andersen^1^, Federico Serrano-Sánchez^1^, Neven Biskup^3,4^, Olivier Mathon^2^, Norbert M. Nemes^1,3^, José L. Martínez^1^, José A. Alonso^1,*^

**Table S1.** Structural parameters of GdMn_2_O_5_ results refined from SXRD data at room temperature (300 K).

| Crystal data, incident radiation of λ = 0.35449 Å | | | | | | |  | |
| --- | --- | --- | --- | --- | --- | --- | --- | --- |
| Orthorhombic *Pbam*  *T* = 300 K |  | $\boldsymbol{a}$ **(Å)** | $\boldsymbol{b}$ **(Å)** | $\boldsymbol{c}$ **(Å)** | $\boldsymbol{\alpha= \gamma=}$ $\boldsymbol{\beta}$ **(º)** | $\boldsymbol{V}$ **(Å^3^)** | | ***Z*** |
|  |  | 7.3582(1) | 8.5452(9) | 5.6860(5) | 90 | 357.527(5) | | 4 |
| Reliability factors |  | ***R*_p_ (%)** | ***R*_wp_ (%)** | ***R*_exp_ (%)** | ***R*_Bragg_ (%)** | **χ^2^** |  | |
|  |  | 4.42 | 5.34 | 3.67 | 2.75 | 3.01 |  | |
| Fractional atomic coordinates and isotropic displacement parameters (Å^2^) | | | | | | |  | |
| Atom | **Site** | ***x*** | ***y*** | ***z*** | ***U* (Å^2^)** | **Occ. (≤ 1)** | | |
| Gd | *4g* | 0.1398(3) | 0.1720(3) | 0 | 0.006 | 1.0 | | |
| Mn1 | *4f* | 0 | 0.5 | 0.257(1) | 0.004 | 1.0 | | |
| Mn2 | *4h* | 0.4108(8) | 0.3504(8) | 0.5 | 0.006 | 1.0 | | |
| O1 | *4e* | 0 | 0 | 0.276(5) | 0.008 | 1.0 | | |
| O2 | *4g* | 0.158(4) | 0.447(3) | 0 | 0.011 | 1.0 | | |
| O3 | *4h* | 0.153(4) | 0.434(3) | 0.5 | 0.007 | 1.0 | | |
| O4 | *8i* | 0.398(3) | 0.207(2) | 0.248(3) | 0.011 | 1.0 | | |

**Table S2.** Structural parameters extracted from EXAFS fitting at Mn *K*- and Gd *L*_3_-edges at 10 K, including the scattering path, coordination number ($N_{i}$), mean pair distance from EXAFS ($R_{EXAFS}$), from XRD ($R_{XRD}$), and bond-length variance ($\sigma_{i}^{2}$).

| Edge | Shell | $\boldsymbol{N}_{\boldsymbol{i}}$ | $\boldsymbol{R}_{\boldsymbol{EXAFS}}\boldsymbol{(}\mathbf{Å}\boldsymbol{)}$ | $\left\langle\boldsymbol{R}_{\boldsymbol{XRD}} \right\rangle\boldsymbol{(}\mathbf{Å}\boldsymbol{)}$ | $\boldsymbol{\sigma}_{\boldsymbol{i}}^{\boldsymbol{2}}\boldsymbol{(}\mathbf{Å}^{\boldsymbol{2}}\boldsymbol{)}$ |
| --- | --- | --- | --- | --- | --- |
| Mn *K* |  |  |  |  |  |
|  | Mn–O | 5.5 | 1.905(7) | 1.913 | 0.0019(6) |
|  | MnꞏꞏꞏMn_1_ | 3 | 2.888(1) | 2.841 | 0.0113(3) |
|  | MnꞏꞏꞏGd | 5 | 3.334(6) | 3.326 | 0.0045(4) |
|  | MnꞏꞏꞏMn_2_ | 4 | 3.496(2) | 3.468 | 0.0168(2) |
| Gd *L*_3_ |  |  |  |  |  |
|  | Gd–O_1_ | 5 | 2.356(6) | 2.359 | 0.0027(2) |
|  | Gd–O_2_ | 3 | 2.462(3) | 2.412 | 0.0027(2) |
|  | GdꞏꞏꞏMn | 6 | 3.333(4) | 3.326 | 0.0025(3) |
|  | GdꞏꞏꞏGd | 2 | 3.892(6) | 3.913 | 0.0007(2) |

**Figure S1.** Isotherms magnetization *M*(*H*) at different temperatures between 1.8 and 300 K.

**Figure S2.** (a) *M*(*H*) curves at different temperatures between 2-62 K with interval of ∆T = 3 K. (b) Arrott-plot for *M*(*H*) curves at the same temperature range.

**Figure S3.** Δ*S_M_^max^* and *RCP^max^* behavior dependent on μ_0_H and their respective power-law fittings, yielding *n* = 1.48 and *m* = 1.53, respectively.

**Figure S4.** Temperature dependence of the adiabatic temperature change (Δ*T_ad_*) of GdMn_2_O_5_ under a magnetic field change of 0–8 T, calculated from the measured heat-capacity data using the constant-entropy method based on the integration of *C_p_*/*T*. A maximum Δ*T_ad_* of approximately 2.6 K is obtained near 12 K.

**Figure S5.** Thermal evolution of the lattice parameters *a*, *b*, and *c*; respectively, determined from SXRD Rietveld refinements between 4 and 300 K.

**Figure S6.** (a) Temperature dependence of the magnetic susceptibility χ(T) measured under a magnetic field of 0.01 T for pressures of 0.07, 0.15, 0.23, 0.48, 0.68, and 0.86 GPa, respectively. (b) First derivative dχ/dT used to determine the Néel temperature T_N_ at each pressure.

**Figure S7.** (a-f) Isothermal magnetization M(H) curves measured at various temperatures between 2 and 64 K (ΔT = 3 K) under applied pressures of 0.07, 0.15, 0.23, 0.48, 0.68, and 0.86 GPa, respectively. The measurements were performed up to 7 T. These curves were used to calculate the magnetic entropy change ΔS_M_ via the Maxwell relation.

**Figure S8.** (a–f) Temperature dependence of the magnetic entropy change –ΔS_M_(T) derived from the M(H) curves shown in Figure S7 for magnetic field changes up to 7 T at each pressure (0.07, 0.15, 0.23, 0.48, 0.68, and 0.86 GPa). The MCE peak temperature T_MCE_ remains essentially constant at ~10 K across all pressures, while the maximum –ΔS_M_ value decreases slightly from ~12.73 to ~11.98 J/kg K as pressure increases.
